# Targeted remodeling of the human gut microbiome using Juemingzi (*Senna* seed extracts)

**DOI:** 10.3389/fcimb.2024.1296619

**Published:** 2024-04-04

**Authors:** Adrienne B. Narrowe, Johanna M. S. Lemons, Karley K. Mahalak, Jenni Firrman, Pieter Van den Abbeele, Aurélien Baudot, Stef Deyaert, Yanfang Li, Liangli (Lucy) Yu, LinShu Liu

**Affiliations:** ^1^ Dairy and Functional Foods Research Unit, Eastern Regional Research Center, Agricultural Research Service, United States Department of Agriculture, Wyndmoor, PA, United States; ^2^ Cryptobiotix SA., Ghent, Belgium; ^3^ Department of Nutrition and Food Science, The University of Maryland, College, Park, MD, United States

**Keywords:** Senna, gut microbiome, Bacteroidota, Enterobacteriaceae, metagenomics, preclinical research, ex vivo, SIFR^®^

## Abstract

The genus *Senna* contains globally distributed plant species of which the leaves, roots, and seeds have multiple traditional medicinal and nutritional uses. Notable chemical compounds derived from *Senna* spp. include sennosides and emodin which have been tested for antimicrobial effects in addition to their known laxative functions. However, studies of the effects of the combined chemical components on intact human gut microbiome communities are lacking. This study evaluated the effects of Juemingzi (*Senna* sp.) extract on the human gut microbiome using SIFR^®^ (Systemic Intestinal Fermentation Research) technology. After a 48-hour human fecal incubation, we measured total bacterial cell density and fermentation products including pH, gas production and concentrations of short chain fatty acids (SCFAs). The initial and post-incubation microbial community structure and functional potential were characterized using shotgun metagenomic sequencing. Juemingzi (*Senna* seed) extracts displayed strong, taxon-specific anti-microbial effects as indicated by significant reductions in cell density (40%) and intra-sample community diversity. Members of the Bacteroidota were nearly eliminated over the 48-hour incubation. While generally part of a healthy gut microbiome, specific species of *Bacteroides* can be pathogenic. The active persistence of the members of the *Enterobacteriaceae* and selected Actinomycetota despite the reduction in overall cell numbers was demonstrated by increased fermentative outputs including high concentrations of gas and acetate with correspondingly reduced pH. These large-scale shifts in microbial community structure indicate the need for further evaluation of dosages and potential administration with prebiotic or synbiotic supplements. Overall, the very specific effects of these extracts may offer the potential for targeted antimicrobial uses or as a tool in the targeted remodeling of the gut microbiome.

## Introduction

The effects of plant-derived functional foods on human health are mediated by interactions with the gut microbiome, so research regarding these interactions can yield knowledge to guide existing usage and to unlock new potential for bioactive compound discovery. *Senna* sp. phytochemicals have long been appreciated in many cultures for their medicinal properties. Components of *Senna* sp. plants are broadly used in traditional medicine systems across many cultures in Asia, Africa, and South America. Also known as sicklepods, *Senna* sp. have a complex botanical history, with many species of *Cassia* reclassified into this genus in the family *Fabaceae* (https://plants.usda.gov). Confusion persists at the species level as well, with the specific epithets *tora, obtusifolia, alexandrina*, and *angustifolia* potentially being used to refer to the same plant as used in traditional medicines. Uses have been found for most parts of the plant, including leaves, seedpods, seeds, and roots (Ali et al., 2021; Stompor-Gorący, 2021; Alshehri et al., 2022). While primarily used for medicinal purposes, plant material is also used as a protein source (Harper and Collins, 1992).

As *Senna* sp. preparations are consumed, they both affect and are affected by the gut microbiome. The Traditional Chinese Medicine product Juemingzi is the seeds of *Senna obtusifolia* or *Senna tora* These seeds are rich in flavonoids and anthraquinones which are among the main compounds previously described in *Senna* sp. preparations (typically teas) are emodin, aloe-emodin, rhein, chrysophanol, and sennosides (Alshehri et al., 2022). Sennosides and emodin are implicated in the strong laxative effects of *Senna* sp. plant components. The clinical importance of sennosides has led to the inclusion of Senna on the World Health Organization Model List of Essential Medicines (2023). The metabolism and potential bioconversion of these compounds begin within the gut, mediated by the gut microbiome (Wang et al., 2021). Mastumoto et al. demonstrated the ability of selected lactic acid bacteria to deglycosylate sennosides *in vivo* in mice which led to increased intestinal motility (Matsumoto et al., 2012) and such microbial conversion of compounds such as sennosides to the aglycone anthraquinone form appears to be necessary to induce the laxative effect of these compounds (Hardcastle and Wilkins, 1970; Zu et al., 2018; Zhao et al., 2022; Zhang et al., 2023) whereupon the active compounds stimulate motility and draw fluid into the colon (Portalatin and Winstead, 2012).

In addition to their accepted laxative effects, *Senna* sp. compounds may possess other medical benefits. The impact of sennosides on the gut microbiome have been studied in models of diabetes and obesity (Wei et al., 2020). Prior research has also considered the antimicrobial bioactivity of *Senna* sp. compounds, including from leaves (Yuan et al., 2020; Zhang et al., 2020; Zeyue et al., 2022). However many studies primarily focused on the effects of individual chemical components on microbial isolates (Chukwujekwu et al., 2006; Mabwi et al., 2023), or synthetic microbial consortia (Mabwi et al., 2023) rather than taking a holistic view of the interaction of these plant products with a complete human gut microbial community. This is an important consideration as actual traditional formulations using *Senna* sp. will include multiple chemical components beyond only emodin or sennosides. For example, traditional preparations may include prebiotic components which may partially mitigate the effects observed in testing of individual compounds (Zu et al., 2018), and the preparation method may have important consequences for the bioavailability of compounds and the severity of their effects (Fu et al., 2018). From the microbial perspective, studies using only isolates or constructed microbial communities risk missing community-level effects which may amplify or compensate for the effects of products as observed for isolates.

To account for the combinatorial effects of multiple chemical compounds and multiple members of the gut microbial community, we evaluated the effects of extracts of *Senna* sp. seeds on complete gut microbial communities using an *ex vivo* incubation system. We measured overall community growth and fermentative outputs and coupled this with shotgun metagenomic characterization of the microbial community structure and functional potential. We found that *Senna* sp. seed extracts have clear and targeted effects on the gut microbial community which can help inform their use.

## Materials and methods

### Preparation of Senna seed extract

Juemingzi was obtained as the retail preparation 决明子 (*Senna* seed), a material used in traditional Chinese medicine and tea. To prepare the extracts, the seeds were ground into a particle size < 40 mesh with a micromill (Bel-Art Products, Pequannock, NJ). Ground seeds were subjected to defatting using a conventional solid-liquid extraction method with hexane as solvent, at a sample to solvent ratio of 1:5 (*w/v*) in an Erlenmeyer flask for 2 h. Subsequently, the mixture underwent gravity filtration to remove the solvent. The defatted Juemingzi powder was left in the fume hood at room temperature overnight to ensure complete removal of any residual hexane. Then, the defatted powder was extracted with 95% ethanol using Soxhlet extractor, and the solvent was removed by a rotatory evaporation. The solid residues were ground with a micromill (Bel-Art Products, Pequannock, NJ) to a fine powder and kept in a -20 °C freezer for future study.

### Fecal incubations, bacterial cell counts, and measurement of fermentation parameters

Fecal samples were collected with prior consent from six healthy adult donors (3 male, 3 female) aged 29-40 per IRB approval from the Ethics Committee of the University Hospital Ghent, Belgium (BC-09977). Exclusion criteria included no antibiotic, prebiotic or probiotic use within 3 months preceding donation. Incubations were conducted using the *ex vivo* SIFR^®^ technology (Cryptobiotix, Belgium) (Van den Abbeele et al., 2023) for 48 hours. For each of the six donors, samples were collected representing the inoculum, media-only incubation, and incubations containing 3g/L of Juemingzi extract. This dosage represents a high-end dose relative to human usage and permitted exploration of the extent of potential effects of these extracts. The background medium used across all incubations was medium M0003 (Cryptobiotix, Ghent, Belgium).

Total bacteria cell counts were determined as described in (Van den Abbeele et al., 2023). Briefly, SYTO 16 stained cells were counted using a BD FACS Verse flow cytometer (BD, Erembodegem, Belgium) and analyzed using FlowJo v. 10.8.1.

The pH of the cultures was measured using a Senseline pH meter F410 (ProSense, Oosterhout, Netherlands.) Headspace gas pressure measurements were conducted at 0 and 48 hours. Short chain fatty acids including acetate, propionate, butyrate and valerate (SCFA) and branched-chain fatty acids (bCFA) which includes the sum of isobutyrate, isocaproate, and isovalerate were determined. SCFA and bCFA were extracted using diethyl ether following a lab protocol previously described (De Weirdt et al., 2010) using GC with flame ionization detection (Trace 1300, Thermo Fisher Scientific, Merelbeke, Belgium).

### DNA extraction, library preparation and sequencing

DNA extraction, shotgun metagenomic library preparation and sequencing were conducted by CosmosID (Germantown, MD, USA.) DNA was isolated using the QIAGEN DNeasy PowerSoil Pro Kit, according to the manufacturer’s protocol (QIAGEN, Germantown, MD, USA.) Extracted DNA samples were quantified using Qubit 4 fluorometer and Qubit™ dsDNA HS Assay Kit (Thermofisher Scientific, Waltham, MA, USA.) DNA sequencing libraries were prepared using the Nextera XT DNA Library Preparation Kit (Illumina, San Diego, CA, USA) and IDT Unique Dual Indexes with total DNA input of 1ng. Genomic DNA was fragmented using a proportional amount of Illumina Nextera XT fragmentation enzyme. Unique dual indexes were added to each sample followed by 12 cycles of PCR to construct libraries. DNA libraries were purified using AMpure magnetic Beads (Beckman Coulter, Indianapolis, IN, USA) and eluted in QIAGEN EB buffer. DNA libraries were quantified using Qubit 4 fluorometer and Qubit™ dsDNA HS Assay Kit. Libraries were then sequenced on an Illumina HiSeq X platform 2x150bp to a target depth of ~3M read pairs per sample.

### Read-based taxonomic and functional profiling

Raw reads were preprocessed by adapter removal and quality trimming using BBDuk v.38.7 from the BBTools package (https://sourceforge.net/projects/bbmap/) with parameters: (k=31, hdist=1, ftm=5; qtrim=r, trimq=10). Reads were additionally filtered using BBDuk to remove reads mapping to the human genome (ref 19.) Trimmed, filtered reads were used as input for MetaPhlAn4 v. 4.0.6 (Blanco-Miguez et al., 2023), with the mpa_vOct22_CHOCOPhlAnSGB_202212 database to perform read-based taxonomic assignment and estimation of relative abundance. Taxonomic and abundance profiles for each of the 18 samples were merged and used as a species/sites table for downstream visualization and analysis. Read-based functional profiles were generated using HUMAnN v.3.6 (Beghini et al., 2021) and normalized to CPM. In addition to gene family and reaction level profiling, the profiles were classified as KEGG orthologs, EC numbers, MetaCyc pathways and GO terms. For samples with an ‘unclassified’ fraction following classification, MetaPhlAn v. 3.0.14 (Beghini et al., 2021) was used to check for substantial proportions of viral reads which would not have been classified using MetaPhlAn4.

### Taxon and pathway association testing

To discover specific and significant associations of microbial taxa and functional pathways with the tested product, we used MaAsLin2 (Microbiome Multivariable Associations with Linear Models) (Mallick et al., 2021). For the taxonomic data, we used log transformed data (relative abundance data for taxon, and CPM normalized for functional data) for all 48-hour samples specifying ‘donor’ as random effects and product as fixed effect specifying ‘NSC’ as the reference level according to the per-feature model: feature ~ (intercept) + product + (1 | donor) where feature is either taxon or pathway depending on the dataset. Multiple testing correction was performed using Benjamini-Hochberg method with the method’s default FDR threshold of 0.25, retaining only results with a q-value below 0.05.

### Other analyses and visualizations

Statistical analyses were conducted using R/RStudio (v.4.1.3) using the packages: tidyverse (v.1.3.1) (Wickham et al., 2019), vegan (v.2.6-2) (Oksanan et al., 2022), ape (v.5.6-2) (Paradis and Schliep, 2018).

### Data availability

Raw metagenomic sequencing data are available in the NCBI Sequence Read Archive associated with BioProject PRJNA961974. Individual accession numbers for each of the 18 samples are listed in **Supplementary File 1**.

## Results

### Cell counts and fermentation parameters

**Figure 1:**
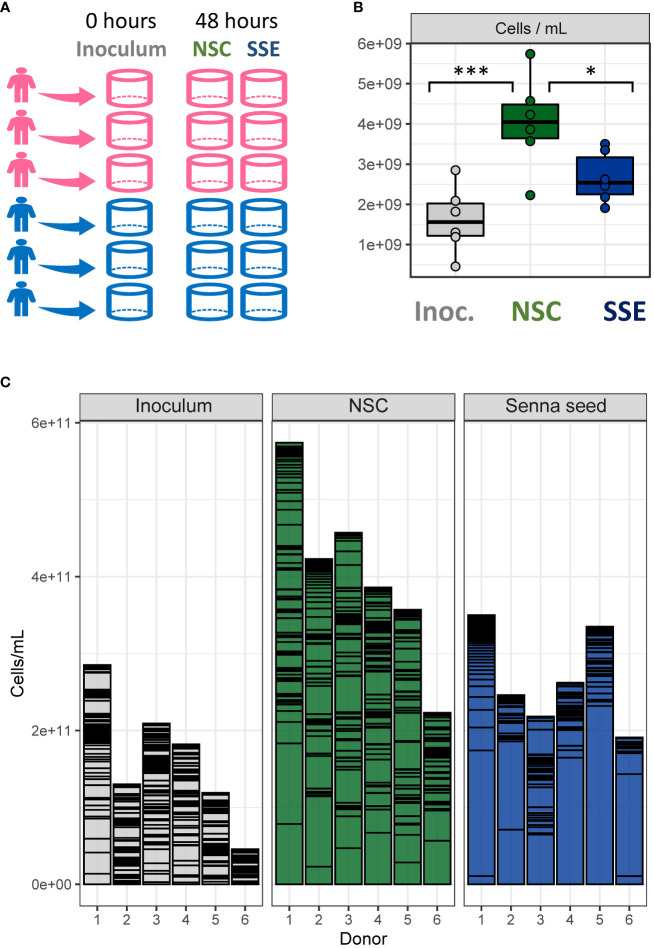
Senna seed extract inhibits microbial growth. **(A)** Experimental design – fecal samples from six donors were incubated for 40 hours as either no-substrate controls (NSC) or with Senna sp. seed extracts (SSE.) **(B)** Total microbial cell counts as determined by flow cytometry reported as cells/mL. **(C)** Total microbial cell counts shown per donor. * = p<0.05, *** = p<0.001.

Total bacterial cell density (cells per mL) ranged from 4.6 x10^8^ to 5.6 x 10^9^ per sample. Cell density did not differ significantly by donor, but the no-substrate control (NSC) samples had significantly higher cell counts than the inoculum, as expected following 48 hours of incubation (two-way ANOVA, Tukey’s HSD, p=0.0009). Unexpectedly, cell numbers in the senna seed extract (SSE) treated samples did not differ significantly from the inoculum. Instead, despite identical incubation times and conditions (**Figure 1A**), the SSE samples had significantly lower cell counts than the NSC (ANOVA, Tukey’s HSD, p=0.047) (**Figure 1B**) Across all treatment groups, cell counts did not differ significantly by donor in any of the treatment groups (**Figure 1C**).

**Figure 2:**
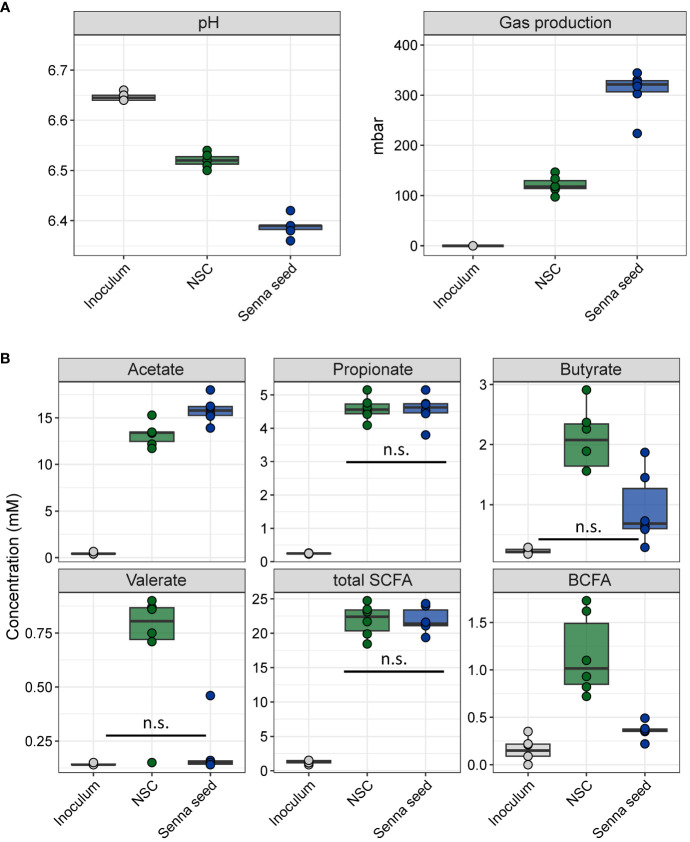
Fermentative end products of senna seed extract incubations **(A)** pH and total gas production **(B)** Short chain and branched chain fatty acid concentrations. All comparisons are significant (ANOVA, Tukey’s HSD test, adjusted p-value <0.05) except for those noted on figure as n.s. n.s., Not significant.

Gas production increased in conjunction with a significant drop in pH from an average of 6.52 in the NSC to 6.39 in the SSE incubations (**Figure 2A**). Except for propionate, all the measured fatty acids differed significantly between the NSC and SSE samples (ANOVA, Tukey’s HSD *post hoc* testing p<0.05) (**Figure 2B**.) BCFA, butyrate and valerate were all lower following SSE treatment relative to NSC samples. Acetate increased from an average of 13.2 mM to 15.8 mM and total gas production more than doubled, going from an average of 120.99 mbar to 307.3 mbar. Since total gas production is a function of community size, it is important to keep in mind that this yield was the result of communities with very low cell density. The increase in gas coupled with a decrease in pH despite modest increases in SCFA concentrations suggests that fermentations occurring in these incubations may yield acidic end products other than the ones we measured in this experiment, or that the CO_2_ generation acidifed the incubations.

### Microbial community diversity

Shotgun metagenomic sequencing yielded an average of 1,506,543 read pairs per sample following read trimming and QC (range 370K to 6.67M), with no significant differences by treatment or by donor. Inoculum samples: mean 853K pairs (+/- 237K, range 370K-996K), NSC samples: mean 1.33M (+/- 722K, range 598K – 2.69M) and SSE samples: mean 2.43M (+/- 2.12M, range 786K – 6.67M) (**Supplementary File 2**).

**Figure 3:**
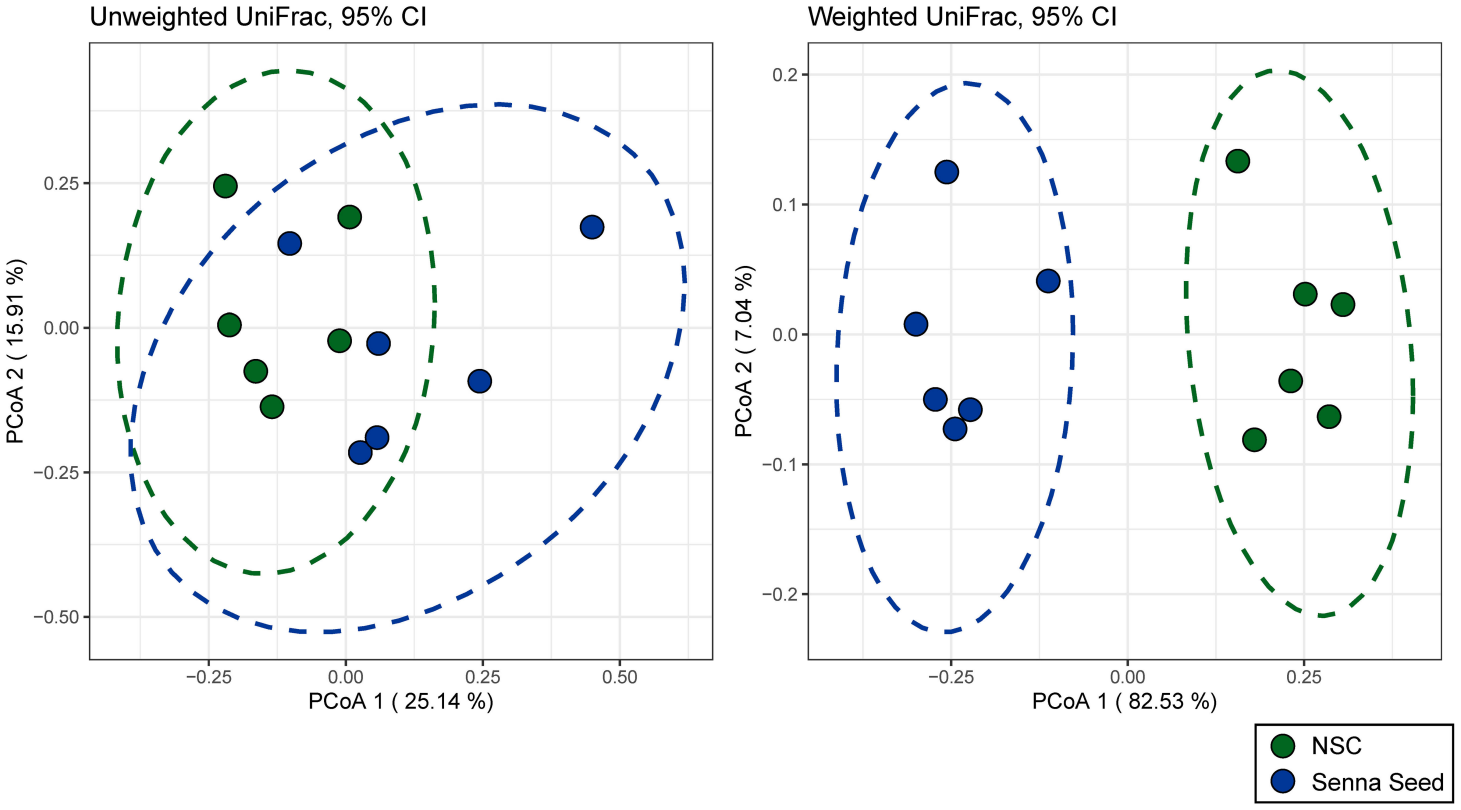
Senna seed extract induces large shifts in beta diversity. Beta diversity as shown by principal coordinates analysis of unweighted (left) and weighted (right) UniFrac distance. Points represent samples and ellipses represent 95% confidence interval.

Using the UniFrac distance metric, we tested if microbial community composition shifted with the addition of SSE (**Supplementary File 3**). Samples clustered significantly by group using both the weighted and unweighted UniFrac metrics (PERMANOVA, p = 0.004 and p = 0.011 respectively) (**Figure 3**), with significant clustering by donor only with the unweighted UniFrac metric (PERMANOVA, p=0.013). The unweighted metric captures overall phylogenetic shifts in community composition, while the weighted metric also incorporates changes in abundance (Lozupone and Knight, 2005; Lozupone et al., 2011) In the case of host-associated microbiomes, inter-individual differences can outweigh small changes associated with treatment (Turnbaugh et al., 2009; Kolodziejczyk et al., 2019; Firrman et al., 2023; Mahalak et al., 2023). However, in this case samples clustered by treatment regardless of donor origin, speaking to the strength of the treatment effects observed. Taken together these data indicate that both the community membership and abundance profiles shifted significantly with SSE treatment.

**Figure 4:**
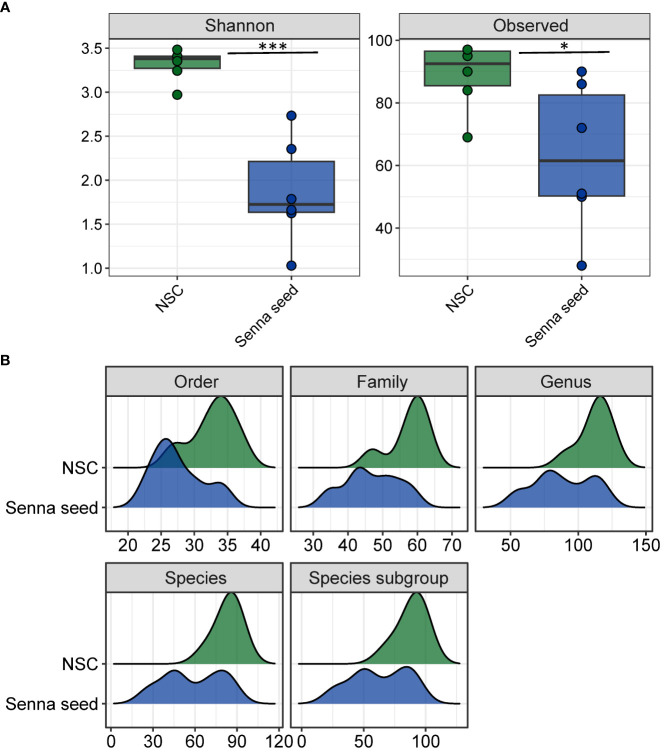
Senna seed extracts reduce microbial community diversity. **(A)** Alpha diversity as measured by Shannon’s diversity index and number of observed taxa. **(B)** Distributions of numbers of taxa observed in NSC and SSE samples for five taxonomic levels. * = p<0.05, *** = p<0.001.

Using the MetaPhlAn4 estimated community composition, we calculated Shannon’s diversity and numbers of observed taxa (richness) as measures of intra-sample alpha diversity (**Figure 4A**). After the 48-hour incubation, community diversity was strikingly and significantly decreased by the treatment with the Senna seed extract (*p* < 0.001 for Shannon’s diversity index). Much of this decrease in diversity may be attributed to a loss of taxa with a drop from an average of 89 taxa in the inoculum and NSC samples respectively, to only 63 taxa in the Senna extract treatment (p = 0.07) (**Figure 4B**, ‘Species subgroup’). The finding of no net growth in the treated incubations coupled with the reductions in diversity indicates that the SSE exerted a bacteriostatic or bactericidal effect on the fecal microbial communities in the incubations. The reductions in numbers of taxa detected at multiple phylogenetic levels indicates that SSE likely restricted the phylogenetic range to a limited number of taxa rather than generating proportional across-the-board reductions in cell numbers. As described below, this is supported by the fact that the taxa which disappeared were not only those which were low in abundance in the NSC which could indicate that they had simply fallen below the level of detection, rather that well-represented taxa were eliminated by SSE in a taxon-specific manner.

### Relative and estimated absolute abundances of community members

**Figure 5:**
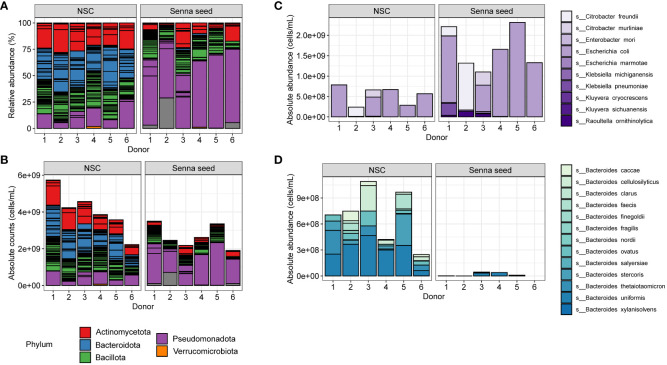
Phylogenetically differentiated effects of Senna seed extracts. **(A)** Phylum level relative abundances in NSC, and SSE samples (L to R). For samples that do not sum to 100% the remainder was unclassified. **(B)** Estimated absolute abundances of phylum level groups arranged as in panel **(A, C)** Estimated absolute abundances of the family *Enterobacteriaceae* for each donor in each condition. Stacked bars are colored to indicate species. **(D)** Estimated absolute abundances of the genus *Bacteroides* for each donor in each condition. Stacked bars are colored to indicate species.

The community shifts underlying the divergence of the SSE samples from the NSC samples as seen in the analysis of alpha and beta diversity measures were clearly visible upon examination of the sample community profiles. At the phylum level, in the SSE treated samples, there is massive and significant shift toward the Pseudomonadota (formerly Proteobacteria) which reached an average of 63% of the community relative abundance (**Figure 5A**) Bacteroidota (formerly Bacteroidetes) was nearly completely eradicated, dropping to an average of 1.3% and this effect was observed for all donors (ranging from 0 to 3.3% of relative abundance.) Average proportions of the Actinomycetota (formerly Actinobacteria) were similarly reduced to 11% (ANOVA, Tukey’s HSD adjusted p < 0.0001). While overall relative abundances of the phylum Bacillota (formerly Firmicutes) did not differ significantly between controls and SSE, not all constituent taxa were unaffected as there were observed losses in members of the family *Oscillospiraceae*, and the *Lachnospiraceae* genera *Dorea* and *Lacrimispora.*

The large increase in Pseudomonadota coupled with the near total loss of Bacteroidota was the most remarkable compositional change associated with SSE. Within the Pseudomonadota, members of the Alpha, Beta, and Deltaproteobacteria classes were present, however none of these classes reached even 1.5% of the community relative abundance. Instead, this large shift in community membership was due to the increases in the abundance of Gammaproteobacteria, specifically members of the family *Enterobacteriaceae*, which comprised from 50 -70% of the SSE communities depending on donor. At the genus to species level, inter-individual differences in the dominant taxa were apparent with *Escherichia coli* comprising the dominant taxon in all donors except Donor 2 which was dominated instead by *Citrobacter freundii* (**Figure 5C**). After 48 hours, members of the Bacteroidota phylum, comprised at most 3.3% of community relative abundance. At the species level, within any donor, only *Bacteroides uniformis* surpassed 1% in any sample (**Figure 5D**), with *Phocaeicola vulgatis*, *Parabacteroides_merdae*, and *Alistipes putredinis* among the most abundant survivors within this phylum, though at abundances < 0.7% in all donors. Taxon losses in the Actinomycetota included the genus *Bifidobacterium* which decreased with SSE treatment from 21% to 10% (ANOVA, Tukey’s HSD adjusted p-value of 0.0039). Of the Bacillota, the families *Lachnospiraceae* and *Oscillospiraceae* (formerly *Ruminococcaceae*) persisted best in the SSE samples, though even within these families, certain taxa fared better than others. For example, members of the genus *Dorea* effectively disappeared decreasing from an average of 2 to 0.5%, while the genus *Enterocloster* increased from an average of 1.8 to 2.7%.

**Figure 6:**
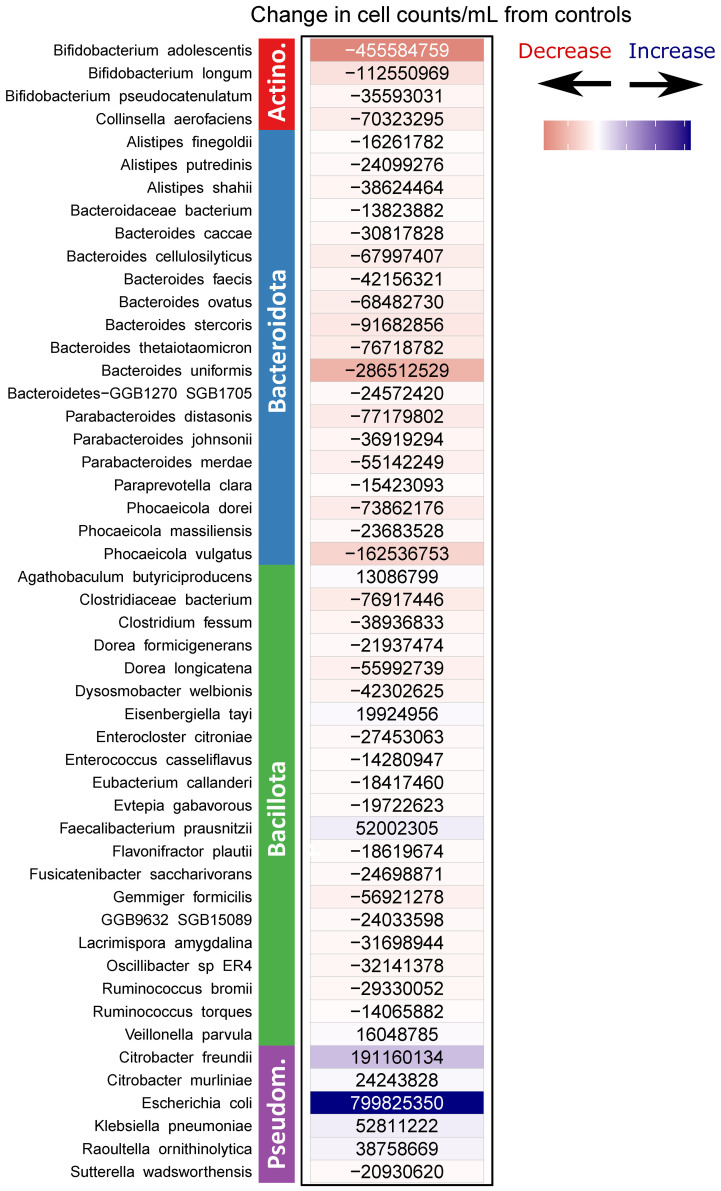
The 50 species with the largest absolute change in across-donor averaged estimated cell counts between NSC and SSE samples. Positive numbers (blue) indicate increases in SSE samples relative to NSC samples. Negative numbers (red) indicate decreases.

**Figures 5B**, **6** shows that these shifts translated to changes in absolute abundance. The 50 most dynamic species, having the largest changes in absolute abundance, were predominantly members of the Bacteroidota (n = 19) and the Bacillota (n = 21), followed by the Pseudomonadota (n = 6) and the Actinomycetota (n = 4). Of these 50 species only nine increased in abundance representing six Pseudomonadota, and three Bacillota_species: *Faecalibacterium prausnitzii, Eisenbergiellatayi*, and *Veillonella_parvula.*

### Changes to microbiome functional metabolic potential

We used Humann3 to perform read-based functional profiling of the metagenomes and annotated these profiles using KEGG orthology and MetaCyc pathway terms, converting estimated functional abundances to counts per million (CPM.) We used these profiles as input to MaAsLin2 to identify genes and pathways differing significantly in the SSE treated samples relative to NSC samples to evaluate if the large community shifts were accompanied by large changes in functional capacity or if the compositionally altered communities benefited from functional redundancy. We observed that many pathways differed significantly in their representation within the SSE communities relative to NSC communities, and in many cases, these gains or losses are of pathways that were contributed either by members of the genus *Bacteroides* or the family *Enterobacteriaceae* (**Supplementary Figure 1**) Importantly, this does not indicate that the pathways were being expressed, only that they were proportionally more or less prevalent in certain samples, however for pathways absent from the community functional repertoire, it is reasonable to expect that those functions were not in fact expressed. The overall consistency of functional shifts with taxonomic shifts suggest that entire functional guilds may have been lost or amplified by SSE.

The MetaCyc pathway, FERMENTATION-PWY: mixed acid fermentation, significantly increased with SSE treatment (~=0.89 log2fold increase; q = 0.02) and correlated significantly with gas production (Pearson r=0.49, p=5e-06), but with neither acetate concentration or pH and only weakly with butyrate (r=-0.22, p=0.044). As gas production outpaced the production of acetate (**Figures 2A, B**), this suggests that not all fermentative outputs of the processes supported by this treatment were measured here. This pathway was primarily contributed by members of the *Enterobacteriaceae* demonstrating how the apparent selectivity of SSE may have yielded altered microbiome function. Additional increases at the pathway level included putrescine biosynthesis (MetaCyc PWY-6305, ~1.4 log2 fold increase, q=0.006), and carnitine degradation (MetaCyc CARNMET-PWY, ~1.6 log2 fold increase, q=0.01), with both pathways largely contributed by the *Enterobacteriaceae* in the SSE samples, demonstrating the realized functional differences associated with the taxonomic shifts. In contrast, the beta-(1,4)-mannan degradation pathway (MetaCyc PWY-7456) was significantly decreased in SSE treated samples relative to controls (~3.0 log2fold decrease, q=0.004). At the gene level (KEGG orthologs), one of the functions most reduced by SSE treatment was butyrate kinase (K00929) (~6.6 log2 fold decrease, q=0.002), a key enzyme in a butyrate production pathway. In the NSC samples, this gene was contributed primarily by *Bacteroides* sp. Also significantly reduced were sialidase (K01186) and alpha L-fucosidase (K01206) genes (~5.5 and ~4.8 log2 fold reductions respectively.) The presence of these genes was also specifically associated with the *Bacteroides* sp. and *Parabacteroides* sp. which disappeared with SSE treatment. Similarly lost were the genes for the *sus* (starch utilization system) operon (K21571 - K21575) (Foley et al., 2016), as with the sialidase and fucosidase genes, the *sus* operon genes were contributed primarily by members of the genus *Bacteroides* (**Supplementary File 4**).

## Discussion

Moving beyond explorations of single chemical constituents on individual members of the gut microbiome, in this study we tested *Senna* sp. extracts of complex composition for their effects on a validated *ex vivo* model of the human gut microbiome. Results showed that in this complex community, in agreement with studies on isolates and synthetic microbial communities, the SSE was highly effective in the selective killing or growth inhibition of certain taxa (Chukwujekwu et al., 2006; Mabwi et al., 2023), namely members of the Bacteroidota. In contrast, and in agreement with those prior studies, bacteria including *Escherichia coli, Citrobacter freundii*, and *Klebsiella pneumoniae* persisted in the presence of SSE and within 48 hours they constituted the major portion of the incubated microbial communities. This is an important expansion on isolate studies as the primary affected groups are known to play important roles in gut health. While these results were consistent with other results testing *Senna* seeds, extracts from *Senna* sp. leaves, when tested in mice yielded a different cohort of bacteria which changed in abundance, suggesting that the part of the plant used is a key determinant of its affects (Yuan et al., 2020; Zhang et al., 2020). Interestingly in once case the abundance of *B. vulgatus* was reported to have increased, however that experiment contained additional variables potentially contributing to the divergent results (Yuan et al., 2020).

Members of the genus *Bacteroides* are obligate fermenters and make up a large proportion of the human gut microbiome (Zafar and Saier, 2021). They have been associated with higher consumption of animal products and fats (Wu et al., 2011) despite their specialization on polysaccharides (Sonnenburg et al., 2005; Zafar and Saier, 2021). The Firmicutes/Bacteroides ratio was long considered metric by which a microbiome composition has been characterized (Turnbaugh et al., 2006; Diener and Gibbons, 2023; Frioux et al., 2023), and reductions in *Bacteroides* spp. has been shown to be a hallmark of healthy aging (Wilmanski et al., 2021). Because of their fermentative capabilities, *Bacteroides* spp. are extensively engaged in bacterial cross-feeding leading to the production of SCFAs, beneficial metabolites which can be used by host cells (Zafar and Saier, 2021). Among them, *B. thetaiotamicron* has been identified as a critical taxon for microbiome community recovery post antibiotics (Chng et al., 2020; Gibbons, 2020). Recent work which revisited the enterotype concept identified ‘enterosignatures’ (ES) and showed that the Bacteroides type (ES-Bact) was metabolically integrated with the Prevotella and Firmicutes types and is important in the development of the gut microbiome (Diener and Gibbons, 2023; Frioux et al., 2023). However, the *Bacteroides* can also be a source of opportunistic infections with *B. fragilis* being a leading pathogen associated with post-surgical polymicrobial infections (Zafar and Saier, 2021) and many *Bacteroides* species exhibit high levels of antibiotic resistance (Wexler, 2007), in fact, the ES-Bact signature increases following antibiotic disruption of the gut microbiome (Diener and Gibbons, 2023; Frioux et al., 2023).

It is unlikely that the drop in *Bacteroides* spp. was due to rapid consumption of the carbohydrates in the growth media as the NSC was able to support a much larger population of these taxa. This implies that the decrease in *Bacteroides* sp. abundance was the result of a specific inhibitory effect of the SSE on members of this genus rather than a starvation effect. Because the relative decrease in the *Bacteroides* coincides with an overall drop in cell counts, it is not likely to be the result of overgrowth of *Enterobacteriaceae*. Rather the dominance of the *Enterobacteriaceae* likely reflects a survival advantage in the face of the components of the SSE, coupled with *Bacteroides*-specific selective toxicity which is not without precedent. In a recent review of the effects of dietary polyphenols on the gut microbiome findings regarding the *Bacteroides* were inconsistent but included evidence indicating that, as in this case, polyphenolic compounds can have specific and targeted effects on the *Bacteroides* (Wang et al., 2022). Additionally, a recent study found that the antipsychotic drug chlorpromazine also selectively inhibited members of the Bacteroidota within a synthetic gut community (Wuyts et al., 2023), and interestingly, the three ring structure of this drug bears some structural similarity to emodin. While the phylogenetic specificity of this effect appears clear, the mechanism remains unknown and will require further study.

These taxonomic trade-offs come with functional shifts in the microbiome that have important implications for host health. The reductions in *Bacteroides* mediated by SSE changed the functional profile of the gut microbiomes in a way which may be detrimental to the host. Loss of *Bacteroides* sp. resulted in the significant reduction in representation of the *sus* operon (starch utilization system) genes. This indicates that the metabolic potential of the residual community may be tilted away from carbohydrate/starch utilization, potentially inducing a real effect on host metabolism and nutrient absorption. Additional functional losses included sialidases, enzymes which facilitate the catabolism of host mucin-derived sialic acids. Fucosidases and sialidases cleave and release the sugars providing substrate for other gut microbial taxa which inhabit the mucosal region. In some cases, these free mucosal-derived sugars can be key substrates for gut bacteria (Pereira et al., 2021; Luis and Hansson, 2023). However, these losses may be less problematic for the host health, as free sialic acids can also aid in the establishment of pathogenic taxa including *C. difficile, Salmonella typhimurium*, or pathogenic *E. coli* (Ng et al., 2013; Tailford et al., 2015; Juge et al., 2016). Additional specific losses include genes associated with butyrate production and beta-(1,4)-mannan degradation genes. As SCFA inhibit inflammation (Morrison and Preston, 2016), and mannans are major components of plant cell hemicelluloses (Moreira and Filho, 2008), the reductions in taxa with these genes indicates an important potential metabolic loss for the host.

Another similar functional loss following SSE addition comes as the result of the decrease in *Bifidobacterium longum.* This bifidobacterial species is a well-known probiotic and was the second most decreased of the *Bifidobacteria* species following SSE treatment (**Figure 6**, **Supplementary Figure 2**). This loss further amplifies the reduction of mucin degrading capacity within the metagenome.) Another bifidobacterial species which decreased in the SSE incubations was *Bifidobacterium pseudocatenulatum.* Interestingly, a strain of *B. pseudocatenulatum*, was shown to be extremely effective at sennoside deglycosylation (Matsumoto et al., 2012). Like other *Bifidobacterium* sp. it is well adapted to the human gut because it possesses carbohydrate active enzymes (CAZymes) including endo-1,4-β-xylanase (Watanabe et al., 2021) and β-glucosidase which is also key in the transformation and activation of sennosides. Losses of these taxa and their repertoire of CAZymes may further reduce the ability of the microbiome to extract energy and produce host-beneficial metabolites from complex polysaccharides.

On the other side of the abundance tradeoff, the family *Enterobacteriaceae* increased in SSE incubations, filling the space vacated by the *Bacteroides* and others. While the overall effect of the SSE was growth inhibitory, the selective survival of these taxa may derive from their ability to better cope with the polyphenolic compounds present in the SSE. The *Enterobacteriaceae* can be associated with dysbiosis and gut inflammation including increased oxygen intrusion (Zeng et al., 2017), though probiotic strains such as *Esherichia coli* Nissle can be found within this family (Pradhan and Weiss, 2020). Incubations in this study were anaerobic, nonetheless, the effects of SSE mimicked the effects sometimes seen in the inflamed gut (Rivera-Chávez et al., 2016), namely increases in the facultative anaerobes, *Enterobacteriaceae*, at the expense of obligate anaerobes of the genus *Bacteroides*. Functional increases in the SSE treated samples primarily associated with the members of this family included putrescine biosynthesis and carnitine. Carnitine use by gut bacteria can ultimately lead to increases in circulating TMAO, a metabolite negatively associated with cardiovascular health (Wang et al., 2011). An enterobacterial outgrowth resulting from SSE could be detrimental so dosages should be tuned to avoid this effect. Additionally, this effect could potentially be mitigated by using strains such as *E. coli* Nissle as a synbiotic in conjunction with SSE which may benefit from the niche space vacated by the *Bacteroides* to prevent overgrowth of potentially detrimental *Enterobacteriaceae* species.

Finally, the persistence of *Faecalibacterium prausnitzii* in the face of this otherwise challenging chemical additive is important. While multiple other members of the Bacillota decreased in both relative and absolute abundance, *F. prausnitzii* increased in abundance relative to controls (**Figure 6**, **Supplementary Figure 2**). *F. prausnitzii* is noted for its ability to produce butyrate (Miquel et al., 2014), fructose (Fagundes et al., 2021) and to generate proteins with host anti-inflammatory effects (Miquel et al., 2015; Quévrain et al., 2016; Lenoir et al., 2020) and members of this taxon have generally been associated with good gastrointestinal health (Bai et al., 2023). Reductions in *F. prausnitzii* have been particularly associated with conditions such as Crohn’s Disease/Ulcerative Colitis and colorectal cancer (Bai et al., 2023). The abundance of *F. prausnitzii* and the ratio of *F. prausnitzii* (Fc) to *E. coli* (Ec) have been suggested as a biomarkers to discern healthy GIT and between GIT disease conditions respectively (Lopez-Siles et al., 2017) In our study, the Fc/Ec ratio decreased from inoculum to incubated samples, but did not differ markedly between controls and treated samples. The exception to this is samples from Donor 2 where *Citrobacter freundii* apparently occupied the niche space otherwise occupied by *E. coli* in the other donors’ samples pointing out a potential weakness in using such a ratio which depends on taxonomic identity while potentially ignoring functionally homologous community members. *F. prausnitzii* has also been associated with *B. thetaiotamicron*, with the former potentially dependent on this *Bacteroides* species to colonize and persist (Wrzosek et al., 2013). If this is the case, it remains an open question how long the *F. prausnitzii* would survive given the stark reduction in *Bacteroides* species abundances, however other research suggested that *E. coli* presence was able to prime a mouse gut model for successful engraftment of *F. prausnitzii* (Miquel et al., 2015) and this pairing is represented in the SSE resistant community. Other Bacillota survivors included the butyrate and lactate producing *Eisenbergiella tayi* (Amir et al., 2014), and the lactate fermenting *Veillonella parvula* which produces acetate and propionate.

Uncontrolled losses of entire phylogenetic groups may represent losses of functional guilds and can have negative effects on the human host if they persist beyond a transient state. While the perceived effects at the host level are short-lived, laxative-mediated short-term disturbances such as these may in fact precipitate persistent effects. In a study of transient osmotic diarrhea induced by PEG laxatives, the humanized murine gut commensal family *Muribaculaceae* (order Bacteroidales) was eliminated with treatment (Tropini et al., 2018). In that study, as here, the Gammaproteobacteria increased during the disturbance period before subsiding. Eventually the lost taxon was able to re-establish, but this required a deliberate intervention in the form of exposure to an environmental reservoir. Prior work by that group demonstrated that *Clostridium difficile* was better able to colonize following PEG disruption (Ferreyra et al., 2014), highlighting the importance of maintaining the stability of a healthy gut microbiome, and the potential risks resulting from this type of disruption.

## Conclusions

Whether the use of *Senna* sp. extracts alone can shift the balance of community in a healthy donor under physiological conditions needs further investigation. The results of this study suggest that for some individuals there may be conditions in which *Senna* sp. extracts or derivative compounds may negatively tip the scales toward an *Enterobacteriaceae*-dominated community with losses in functional capacity to metabolize plant polysaccharides and produce beneficial SCFAs. However, the persistence and growth of anti-inflammatory *Faecalibacterium prausnitzii* offers the hope that an SSE impervious community does exist which can buffer any negative effects of enterobacterial overgrowth. These results clearly indicate that the dosage threshold should be further evaluated to be mindful of these community tradeoffs. Additionally, enterobacterial probiotic strains such as *E. coli* Nissle could be employed in conjunction with *Senna* extracts to potentially further modulate the resulting community composition. While the mechanism of the focused antibacterial effect remains to be elucidated, these results offer a potential avenue for further experimentation on the use of *Senna* sp. extracts as part of a toolkit for remodeling the gut microbiome or for antibacterial purposes, specifically targeting *Bacteroides* species or members of the Bacillota. As certain *Bacteroides* species such as *Bacteroides fragilis* are opportunistic pathogens, and *Clostridium difficile* infection is clinically significant, *Senna* sp. derived compounds may yet find a therapeutic role beyond laxative effects.

## Data availability statement

The datasets presented in this study can be found in online repositories. The names of the repository/repositories and accession number(s) can be found in the article/**Supplementary Material**.

## Ethics statement

The studies involving humans were approved by Ethics Committee of the University Hospital Ghent, Belgium. The studies were conducted in accordance with the local legislation and institutional requirements. The participants provided their written informed consent to participate in this study.

## Author contributions

AN: Data curation, Formal analysis, Visualization, Writing – original draft, Writing – review & editing. JL: Writing – review & editing. KM: Writing – review & editing. JF: Writing – review & editing. PV: Conceptualization, Investigation, Methodology, Supervision, Writing – review & editing. AB: Investigation, Methodology, Writing – review & editing. SD: Investigation, Methodology, Writing – review & editing. YL: Investigation, Resources, Writing – review & editing. LY: Conceptualization, Resources, Writing – review & editing. LL: Conceptualization, Supervision, Writing – review & editing.

## Supplementary material

Supplementary Figure 1 Estimated functional abundances for selected MetaCyc pathways showing contributions by species.

Supplementary Figure 2 Estimated cell counts for the genera *Faecalibacterium* and *Bifidobacterium*, and family *Clostridiaceae*.

Supplementary File 1 NCBI accessions and read counts for raw sequencing data.

Supplementary File 2 Cell counts, SCFA, gas, and pH measurements, and alpha diversity metrics for all samples.

Supplementary File 3 MetaPhlAn taxonomic profile and relative abundances.

Supplementary File 4 Excel file with MaAsLin2 results for MetaCyc pathways (sheet 1) and KEGG orthologs (KOs) (sheet 2).
