# Supplementary figures and images for: Targeted remodeling of the human gut microbiome using Juemingzi (Senna seed extracts)
Source: Front Cell Infect Microbiol. 2024 Apr 4;14:1296619. doi: 10.3389/fcimb.2024.1296619 (PMC11024242; doi:10.3389/fcimb.2024.1296619)

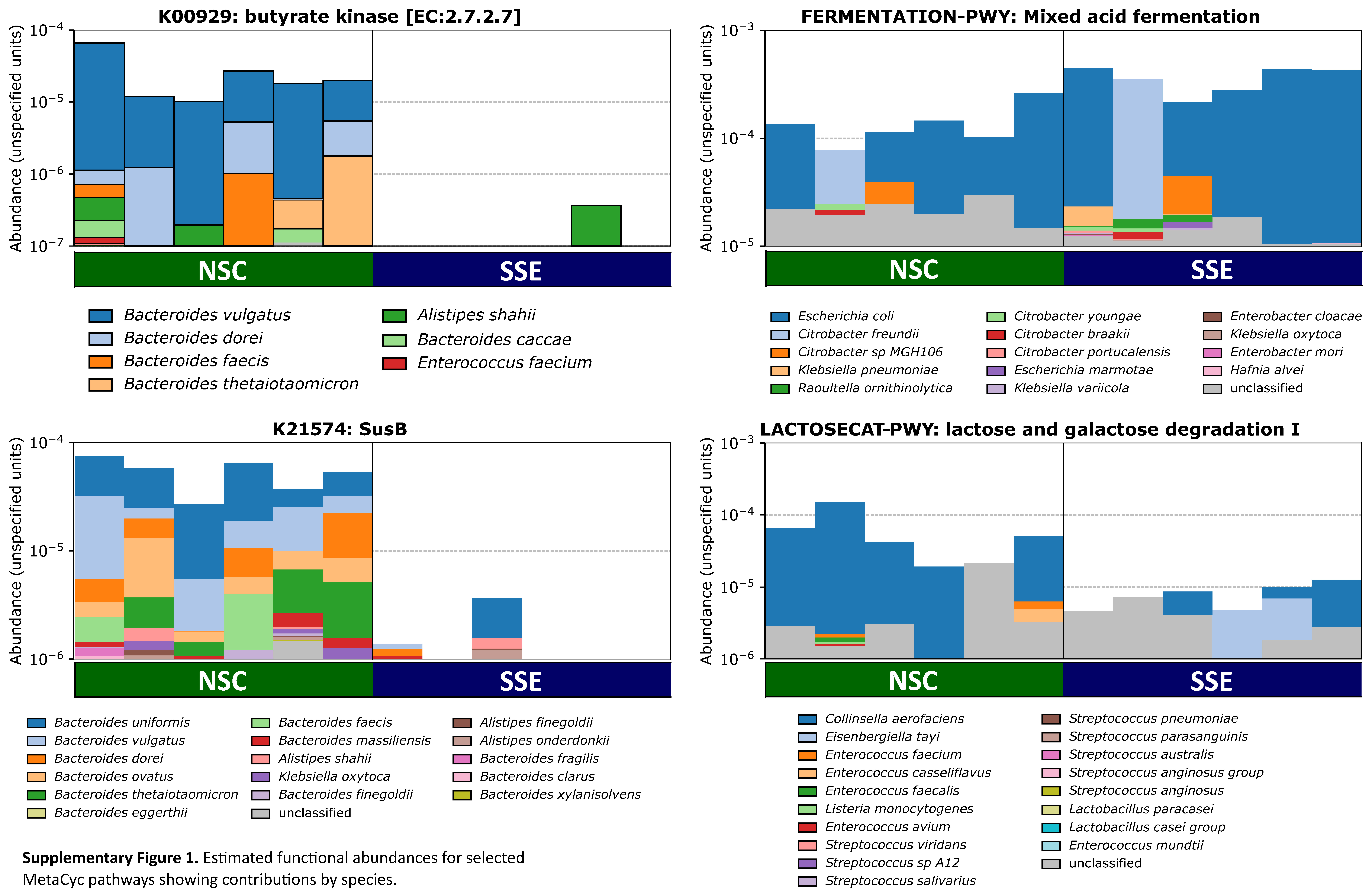

Supplement: Supplementary Figure 1 — Estimated functional abundances for selected MetaCyc pathways showing contributions by species. [file Image_1.jpg]
